# Intravenous thrombolysis is more safe and effective for posterior circulation stroke: Data from the Thrombolysis Implementation and Monitor of Acute Ischemic Stroke in China (TIMS-China)

**DOI:** 10.1097/MD.0000000000003848

**Published:** 2016-06-17

**Authors:** Xu Tong, Xiaoling Liao, Yuesong Pan, Yibin Cao, Chunjuan Wang, Liping Liu, Huaguang Zheng, Xingquan Zhao, Chunxue Wang, Yilong Wang, Yongjun Wang

**Affiliations:** a Department of Neurology, Beijing Tiantan Hospital, Capital Medical University, Beijing; b Department of Neurology, Tangshan Gongren Hospital, Hebei Medical University, Tangshan, Hebei; c China National Clinical Research Center for Neurological Diseases; d Tiantan Clinical Trial and Research Center for Stroke, Department of Neurology, Beijing Tiantan Hospital, Capital Medical University; e Vascular Neurology, Department of Neurology, Beijing Tiantan Hospital, Capital Medical University; f Center of Stroke, Beijing Institute for Brain Disorders; g Neuro-intensive Care Unit, Department of Neurology, Beijing Tiantan Hospital, Capital Medical University; h Department of Neuropsychiatry and Behavioral Neurology and Clinical Psychology, Beijing Tiantan Hospital, Capital Medical University; i Beijing Key Laboratory of Translational Medicine for Cerebrovascular Disease, Beijing, China.

**Keywords:** acute ischemic stroke, anterior circulation, intravenous thrombolysis, outcome, posterior circulation

## Abstract

We aimed to compare the safety and efficacy of intravenous thrombolysis (IVT) with alteplase for anterior circulation stroke (ACS) and posterior circulation stroke (PCS). From a large multicenter prospective registry—the Thrombolysis Implementation and Monitor of Acute Ischemic Stroke in China database—all patients who received IVT within 4.5 hours after stroke onset was reviewed. According to the clinical presentations and imaging findings, the eligible patients were divided into ACS and PCS groups. The safety and efficacy outcome measures included post-IVT symptomatic intracranial hemorrhage (sICH), parenchymal hematoma, and all intracranial hemorrhage (aICH) within 7 days, mortality within 90 days, excellent recovery (modified Rankin Scale 0–1), and functional independence (modified Rankin Scale 0–2) at 90 days. For comparing the outcomes between both groups, the odds ratios (ORs) with 95% confidence intervals (CIs) and the adjusted ORs with 95% CIs were analyzed by univariate and multivariate logistic regression models. Of 953 patients enrolled, 829 patients had ACS and 124 had PCS. The patients with PCS had less often atrial fibrillation (11.3% vs 19.8%; *P* = 0.02), higher blood glucose level (8.31 vs 7.63 mmol/L; *P* = 0.02), and more white blood cell counts (8.79 vs 7.75 × 10^9^/L; *P* = 0.001) than those with ACS. After adjustment for the potential confounders, multivariate logistic analysis showed that PCS patients had not only lower rates of sICH (3.2% vs 7.7%; OR 0.28, 95% CI 0.09–0.90), parenchymal hematoma (1.6% vs 9.2%; OR 0.13, 95% CI 0.03–0.57), and aICH (8.1% vs 20.4%; OR 0.26, 95% CI 0.12–0.54), but also higher proportions of excellent recovery (55.7% vs 41.6%; OR 2.27, 95% CI 1.42–3.61) and functional independence (63.9% vs 53.0%; OR 2.33, 95% CI 1.40–3.89) compared with ACS patients. However, there was no significant difference in the occurrence of mortality (OR 0.86, 95% CI 0.39–1.91) between both groups in the multivariate model, although more PCS patients seemed to die within 90 days than did ACS patients (15.6% vs 10.1%; OR 1.64, 95% CI 0.96–2.82) in the univariate analysis. Our study suggests that IVT with alteplase is more safe and effective for PCS.

## Abstract 2

Supplemental Digital Content is available in the text

## 1 Introduction

Intravenous thrombolysis (IVT) with alteplase is still the first-line therapy for all kinds of acute ischemic stroke (AIS) including anterior circulation stroke (ACS) and posterior circulation stroke (PCS),^[1–3]^ although endovascular treatment recently has achieved the recommendation of class I for carefully selected patients with ACS.^[4]^ In the past 2 decades, several randomized controlled trials and real world registries have demonstrated the safety and efficacy of intravenous alteplase for AIS within 4.5 hours time window.^[5–8]^ Unfortunately, reports on using IVT for PCS are lacking. For example: National Institute of Neurological Disorders and Stroke (NINDS) trial had few PCS patients (5%);^[5]^ European Cooperative Acute Stroke Study (ECASS) I and II only included the patients with ACS, excluding those with PCS;^[9,10]^ ECASS III did not refer to the number of PCS patients if any was enrolled;^[7]^ Safe Implementation of Thrombolysis in Stroke (SITS) was the largest stroke thrombolysis registry in the world, but also did not differentiate the sites of infarction.^[6,8]^ PCS was often overlooked in the previous clinical studies partly because of its low incidence. To be specific, PCS only accounted for 17% to 22% of all AIS in Chinese hospital-based population.^[11,12]^ On the other hand, stroke physicians do not care whether a patient had ACS or PCS at the clinical scene, and thereby PCS is often treated similarly to ACS,^[13]^ but results of anterior circulation trials do not necessarily apply to PCS. To determine whether there is any difference in the post-IVT outcomes between ACS and PCS, we analyzed the data from a large multicenter prospective registry—the Thrombolysis Implementation and Monitor of Acute Ischemic Stroke in China (TIMS-China).

## 2 Methods

### 2.1 Study population

The TIMS-China was a national prospective stroke registry of thrombolytic therapy for patients with AIS in 67 major stroke centers in China.^[14]^ The study protocol was approved by the Ethics Committee of Beijing Tiantan Hospital. The registry was regularly monitored independently by the Quality Monitoring Committee of TIMS-China and the Contract Research Organization. All patients or patients’ care providers were given written informed consents before thrombolysis, and all patients received the alteplase dose of 0.5 to 0.9 mg/kg, with 10% of the total dose given within 1 minute followed by the remainder infused over 60 minutes. The National Institutes of Health Stroke Scale (NIHSS) score was measured at baseline, 2 hours, 24 hours, 7 days (or at discharge, whichever occurs first), and any time of neurological deterioration. The modified Rankin Scale (mRS) score was assessed at 7 days (or at discharge, whichever occurs first) and 90 days. Only the neurologists who were trained and qualified for using NIHSS and mRS recorded the scores. Brain imaging (computed tomography [CT] and magnetic resonance) was performed at baseline, 24 hours, and 7 days (or at discharge, whichever occurs first), but magnetic resonance examination was optional. The imaging findings were interpreted by at least 2 experienced senior radiologists in each participating hospital.

From the TIMS-China database, all patients who received IVT within 4.5 hours time window were screened for this analysis. Based on the clinical presentations and imaging findings, the patients were divided into ACS or PCS group. ACS was defined as acute infarctions involving the territories of internal carotid artery, middle cerebral artery, or anterior cerebral artery. PCS was defined as acute infarctions involving the territories of vertebral artery, basilar artery, or posterior cerebral artery. The patients with unclear stroke territory and acute infarctions in both circulation territories were excluded.

### 2.2 Safety and efficacy outcomes

The safety outcomes included post-IVT symptomatic intracranial hemorrhage (sICH), parenchymal hematoma (PH), and all intracranial hemorrhage (aICH) within 7 days, and mortality within 90 days. sICH was evaluated by using the NINDS definition, which was defined as hemorrhage that was not seen on a previous CT scan, and there was subsequently either a suspicion of hemorrhage or any decline in neurological status.^[5]^ Parenchymal hematoma (PH) was defined as a hemorrhage with mass effect: PH1, blood clots not exceeding 30% of the infarcted area with slight space-occupying effect; PH2, blood clots exceeding 30% of the infracted area with substantial space-occupying effect.^[10]^ aICH was verified by the follow-up imaging regardless of clinical deterioration.

The efficacy outcomes included excellent recovery and functional independence at 90 days. Excellent recovery was defined as having a mRS score of 0 to 1, and functional independence was defined as having a mRS score of 0 to 2.^[8]^

### 2.3 Statistical analysis

The baseline data were compared between ACS and PCS groups. The *t* test or the Mann–Whitney *U* test was used to compare means or medians for continuous variables. The Pearson chi-square test or continuity correction was used to compare the proportions for categorical variables. For comparing the post-IVT outcomes between both groups, odds ratios (ORs) with 95% confidence intervals (CIs) and the adjusted ORs with 95% CIs were calculated by using univariate and multivariate logistic regression models. The stroke territory (ACS or PCS) was forced in both models as an independent variable. Age, sex, baseline NIHSS score, and the baseline variables showing possible associations with the outcomes in the univariate analysis (*P* < 0.05) were entered in the multivariate model as confounding factors. Statistical significance was set at *P* < 0.05. All analyses were performed with SAS statistical software (version 9.3, SAS Institute Inc., Cary, NC).

## 3 Results

**Figure 1:**
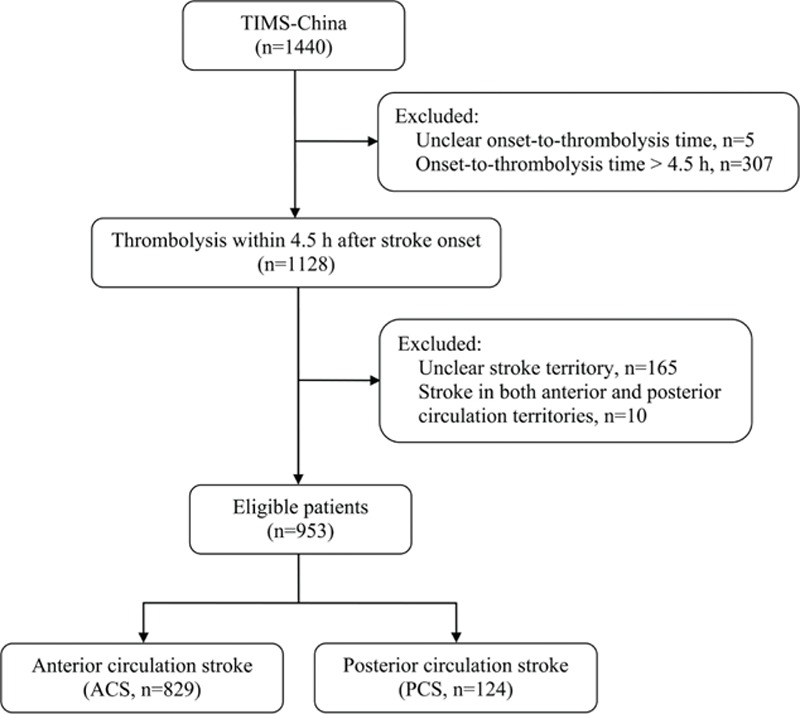
Flow chart of eligible patients. Baseline characteristics of the eligible patients in this study (n = 953) were compared with those of the patients excluded from this study because of unclear stroke territory (n = 165). No differences in demographic and clinical characteristics were identified between the 2 groups, except for hypertension, baseline NIHSS score, and ischemic stroke subtypes. More details are shown in Table S1. TIMS-China = Thrombolysis Implementation and Monitor of Acute Ischemic Stroke in China.

Between May 2007 and April 2012, 1440 patients with AIS and received IVT with alteplase were registered in the TIMS-China database. Because of delayed treatment (>4.5 hours) and unclear onset-to-thrombolysis time, 312 patients were excluded. Another 165 patients with unclear stroke territory and 10 patients with acute infarctions in both anterior and posterior circulation territories were excluded. Finally, 953 eligible patients were entered into the analysis in this study, which included 829 patients in the ACS group and 124 in the PCS group (Fig. 1).

### 3.1 Baseline characteristics

The PCS group had less atrial fibrillation (11.3% vs 19.8%; *P* = 0.02), but higher blood glucose level (mean 8.31 vs 7.63 mmol/L; *P* = 0.02) and elevated white blood cell counts (mean 8.79 vs 7.75 × 10^9^/L; *P* = 0.001) than the ACS group. Whereas other baseline variables, including age, NIHSS score, onset-to-thrombolysis time, and so on, did not have significant differences between the 2 groups (*P* > 0.05) (Table 1).

**Table 1:**
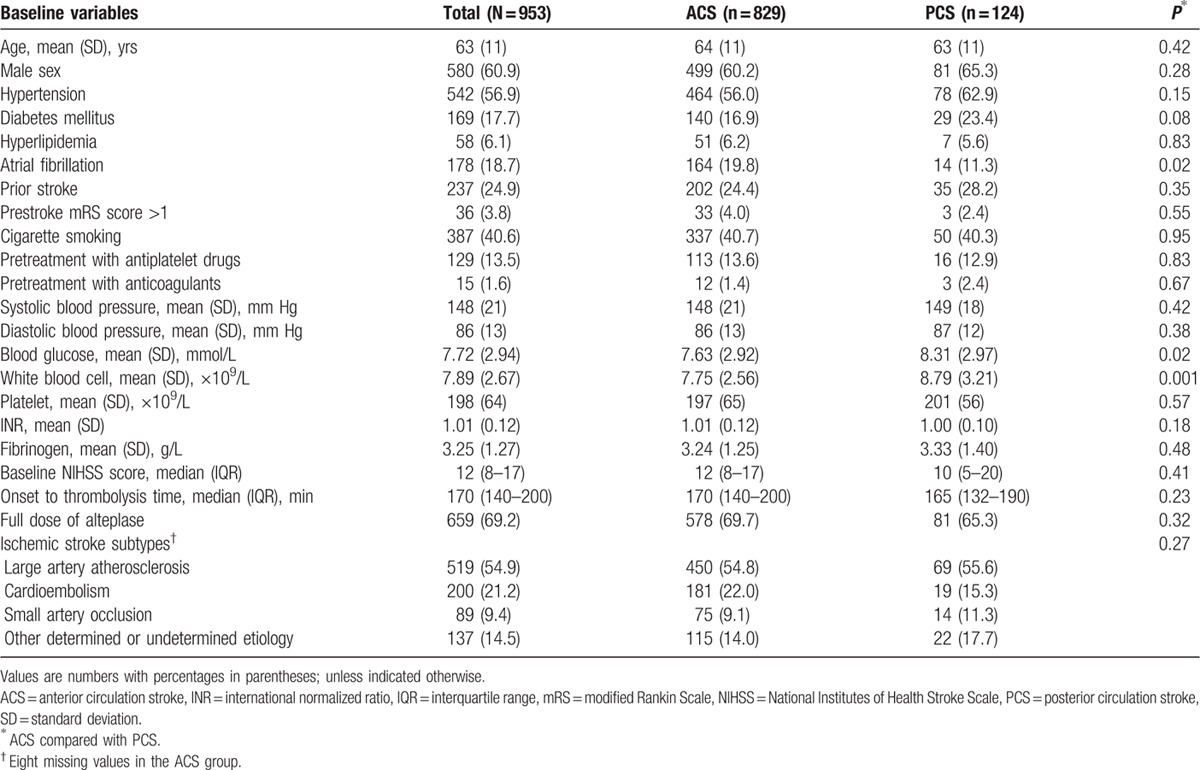
Baseline characteristics of ACS and PCS patients.

### 3.2 Safety and efficacy outcomes

**Figure 2:**
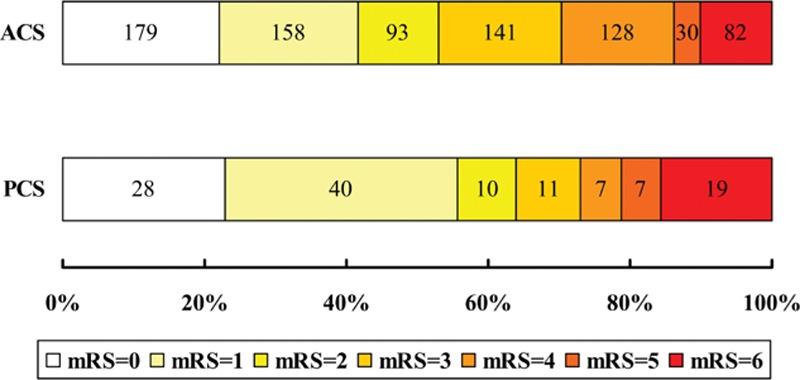
The distribution of mRS at 90 days among ACS and PCS patients. ACS = anterior circulation stroke, PCS = posterior circulation stroke, mRS = modified Rankin Scale.

Twenty patients (2.1%) were lost to follow-up (18 cases with ACS and 2 with PCS) at 90 days. The multivariate logistic regression analysis showed that PCS patients had less events of sICH (3.2% vs 7.7%; OR 0.28, 95% CI 0.09–0.90, *P* = 0.03), PH (1.6% vs 9.2%; OR 0.13, 95% CI 0.03–0.57, *P* = 0.01), and aICH (8.1% vs 20.4%; OR 0.26, 95% CI 0.12–0.54, *P* < 0.001) than ACS patients. In addition, the odds of having both excellent recovery (55.7% vs 41.6%; OR 2.27, 95% CI 1.42–3.61, *P* = 0.001) and functional independence (63.9% vs 53.0%; OR 2.33, 95% CI 1.40–3.89, *P* = 0.001) were approximately 1.3 times more in the PCS group than in the ACS group. However, there was no significant difference in the rate of mortality (OR 0.86, 95% CI 0.39–1.91, *P* = 0.72) between the 2 groups after adjusting the prespecified confounders, although PCS patients were more likely to decease within 90 days than ACS patients (15.6% vs 10.1%; OR 1.64, 95% CI 0.96–2.82, *P* = 0.07) in the univariate analysis (Table 2 and Fig. 2).

**Table 2:**
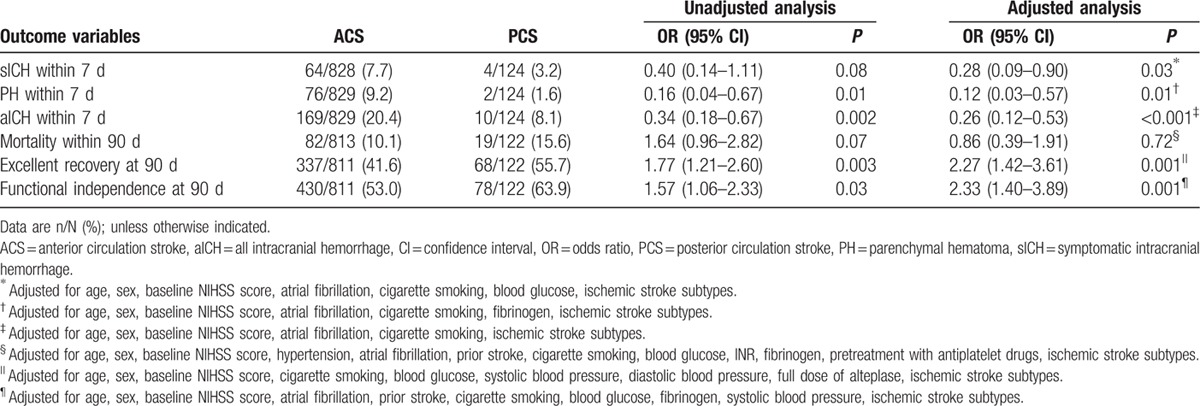
Outcomes of ACS and PCS patients.

## 4 Discussion

The results of this study ran parallel to the previous studies which reported that PCS was associated with a lower risk of hemorrhagic transformations.^[15–18]^ Older age, hyperglycemia, and high NIHSS score are related to poor outcome of post-IVT in all parameters (intracranial hemorrhage, mortality, and independence). High systolic blood pressure and atrial fibrillation are additional predictors of intracranial hemorrhage.^[19]^ In this study, the age, baseline NIHSS score, and systolic blood pressure were similar between ACS and PCS groups. Blood glucose level was higher in the PCS group, whereas atrial fibrillation was less frequent in the PCS patients, potentially affecting the incidence of hemorrhagic transformations in favor of ACS patients. Because the infarction volume on baseline imaging could predict the risk of post-IVT intracranial hemorrhage,^[20,21]^ the smaller infarction volume in PCS compared with ACS might also contribute to the lower rate of hemorrhagic transformations in PCS patients.^[22]^ In addition, the collateral supply in posterior circulation territory might be better than that of anterior circulation.^[23]^ Patients with better collaterals were not prone to having hemorrhagic complications after acute reperfusion therapy.^[24,25]^ Moreover, the brain histopathological changes after stroke onset may be different between both circulation territories. Previous studies have suggested a delayed blood–brain barrier disruption in posterior circulation compared with anterior circulation.^[26,27]^ The greater ischemic tolerance in posterior circulation may partly explain the decreased risk of hemorrhagic complications in PCS.^[28]^

Inconsistent with previous studies,^[16,29–31]^ our results showed that PCS patients had better responses to alteplase and thereby they had higher odds of excellent recovery and functional independence at 90 days than ACS patients. Possible reasons are as follows: the most devastating stroke—basilar artery occlusion—is seldom seen, only accounting for 8% of PCS.^[32]^ Often PCS locates in the cerebellum, hippocampus, or occipital lobe, whereas the brain stem or thalamus is spared. These patients with vertigo, ataxia, impaired vision, cognitive decline, or mental disorder may not have obvious neurological deficits after medical treatment and rehabilitation.^[33]^ On the other hand, because PCS patients had less often hemorrhagic complications in this cohort, they were more likely to be treated by antiplatelet agents or anticoagulants after IVT without fear of bleeding expansion. The subsequent antithrombotic therapies could consolidate the thrombolytic effect and have a positive impact on the outcome.

Our study had several limitations. Firstly, the design of this study is prospective observational cohort by nature. We presented adjusted OR as final results in multivariate logistic regression analyses. However, the confounding factors may not be completely removed by using the multivariate model. In addition, there may be some hidden confounders (e.g., volume of infarction and collateral circulation) we did not collect in this study. We should be careful to interpret the results. Secondly, the sample size was relatively small, especially in the PCS group, which could have reduced the power of test. Thirdly, we had no information about fetal origin of posterior cerebral artery. These patients could have a stroke from the anterior circulation. And finally, this study was conducted in Chinese population. However, ethnic differences may have an impact on the outcomes observed in this analysis. Our findings should be interpreted with caution and could not easily be extrapolated to other populations.

To the best of our knowledge, this was the first multicenter observational study comparing the safety and efficacy of IVT for ACS and PCS in Chinese population. Our study suggested that PCS patients treated with IVT had a lower risk of developing hemorrhagic transformation within 7 days and better chance of having no major disability at 90 days than ACS patients. In short, IVT might be more safe and effective for PCS. Our results will provide reassurance to the clinicians in using intravenous alteplase to treat all kinds of stroke patients with confidence, including those with PCS.

## Supplementary Material

Supplemental Digital Content
