# Supplementary material for: Intravenous thrombolysis is more safe and effective for posterior circulation stroke: Data from the Thrombolysis Implementation and Monitor of Acute Ischemic Stroke in China (TIMS-China)
Source: Medicine (Baltimore). 2016 Jun 17;95(24):e3848. doi: 10.1097/MD.0000000000003848 (PMC4998451; doi:10.1097/MD.0000000000003848)
Supplement: Supplemental Digital Content [file medi-95-e3848-s001.doc]

**Table S1. Baseline characteristics of patients in this study and those with unclear stroke territory**

| **Baseline variable** | **Patients in this study**  **(n=953)** | **Patients with unclear stroke territory**  **(n=165)** | **P value** |
| --- | --- | --- | --- |
| Age, mean (SD), years | 63 (11) | 64 (12) | 0.94 |
| Male sex | 580 (60.9) | 104 (63.0) | 0.60 |
| Hypertension | 542 (56.9) | 117 (70.9) | 0.001 |
| Diabetes mellitus | 169 (17.7) | 25 (15.2) | 0.42 |
| Hyperlipidemia | 58 (6.1) | 13 (7.9) | 0.38 |
| Atrial fibrillation | 178 (18.7) | 23 (13.9) | 0.14 |
| Prior stroke | 237 (24.9) | 49 (29.7) | 0.19 |
| Prestroke mRS score > 1 | 36 (3.8) | 6 (3.6) | 0.93 |
| Cigarette smoking | 387 (40.6) | 65 (39.4) | 0.77 |
| Pretreatment with antiplatelet drugs | 129 (13.5) | 24 (14.5) | 0.73 |
| Pretreatment with anticoagulants | 15 (1.6) | 4 (2.4) | 0.65 |
| Systolic blood pressure, mean (SD), mmHg | 148 (21) | 147 (21) | 0.92 |
| Diastolic blood pressure, mean (SD), mmHg | 86 (13) | 85 (12) | 0.72 |
| Blood glucose, mean (SD), mmol/L | 7.72 (2.94) | 7.68 (3.53) | 0.89 |
| White blood cell, mean (SD), ×10^9^/L | 7.89 (2.67) | 7.92 (2.94) | 0.89 |
| Platelet, mean (SD), ×10^9^/L | 198 (64) | 200 (73) | 0.70 |
| INR, mean (SD) | 1.01 (0.12) | 1.05 (0.28) | 0.08 |
| Fibrinogen, mean (SD), g/L | 3.25 (1.27) | 3.21 (1.03) | 0.66 |
| Baseline NIHSS score, median (IQR) | 12 (8-17) | 8 (5-11) | < 0.001 |
| Onset to thrombolysis time, median (IQR), min | 170 (140-200) | 165 (141-185) | 0.11 |
| Full dose of alteplase | 659 (69.2) | 121 (73.3) | 0.28 |
| Ischemic stroke subtypes^*^ |  |  | 0.001 |
| Large artery atherosclerosis | 519 (54.9) | 81 (49.7) |  |
| Cardioembolism | 200 (21.2) | 21 (12.9) |  |
| Small artery occlusion | 89 (9.4) | 27 (16.6) |  |
| Other determined or undetermined etiology | 137 (14.5) | 34 (20.9) |  |

Values are numbers with percentages in parentheses, unless indicated otherwise.

^*^Eight missing values in the ACS group, two missing values in the ACS group.

Abbreviations: mRS=modified Rankin Scale, INR=international normalized ratio, NIHSS=National Institutes of Health Stroke Scale, SD=standard deviation, IQR=interquartile range.
